# Molecular studies on the impact of microbiome on anticancer drug resistance

**DOI:** 10.3389/fimmu.2026.1848345

**Published:** 2026-06-15

**Authors:** Ke Zhou, Ping Lu, Hongli Xu, Ling Wang, Xinjun Liang

**Affiliations:** 1 Department of Abdominal Oncology, Hubei Cancer Hospital, Tongji Medical College, Huazhong University of Science and Technology, Wuhan, China; 2 Hubei Provincial Clinical Research Center for Colorectal Cancer, Wuhan, China; 3 Wuhan Clinical Research Center for Colorectal Cancer, Wuhan, China; 4 College of Biomedicine and Health, Huazhong Agricultural University, Wuhan, China

**Keywords:** anticancer drug resistance, biotransformation, gut microbiome, immunomodulation, microbial metabolites

## Abstract

Cancer remains a leading global health challenge, with drug resistance posing a critical barrier to the durable efficacy of anticancer therapies, including chemotherapy, immunotherapy, and targeted treatments. Growing evidence highlights the gut microbiome as a key modulator in this resistance process. The gut microbiome—a dynamic and diverse microbial ecosystem—can influence drug efficacy through multiple mechanisms, including the biotransformation of chemotherapeutics, modulation of immune responses, alteration of host drug-metabolizing enzymes, and reshaping of the tumor microenvironment. Notably, specific bacterial taxa can enzymatically inactivate chemotherapeutic drugs, while microbial metabolites and signaling pathways further promote resistance by rewiring cellular survival and immune-regulatory programs. In this review, we synthesize recent molecular insights into microbiome-driven anticancer drug resistance and discuss emerging microbiome-targeted strategies, such as dietary intervention, probiotics and prebiotics, fecal microbiota transplantation, and advanced drug delivery systems. A deeper understanding of host-microbiome-drug interactions may provide new opportunities to overcome therapeutic resistance and advance precision oncology.

## 1 Introduction

Over the past decade, gut-microbiome studies have surged to the forefront of life-science research. This microbial ecosystem, composed of trillions of cells and their pooled genomes, has orchestrated host physiology and determines the balance between health and disease (1, 2). The gut microbiome plays a key role in human health, including the regulation of nutrient metabolism, the formation and maturation of the immune system (3), the “shielding effect” against pathogenic microorganisms (4), the impact on mental health by microbiota-gut-brain axis (5), and the absorption of medications (6). Consequently, dysbiosis is associated with chronic disorders ranging from metabolic syndrome to malignancy (4, 7–10). Pre-clinical studies further indicate that gut microorganisms can either promote or suppress tumor development in various tissues, such as colorectal, gastric, and breast cancers (11).

Meanwhile, cancer remains a leading threat to global health, with the International Agency for Research on Cancer (IARC) reporting approximately 20 million new cases and 9.7 million deaths worldwide in 2022 (12). Drug resistance, whether intrinsic or acquired, remains the dominant cause of treatment failure across chemotherapy, targeted therapy, and immunotherapy (13).

Beyond tumor initiation, the microbiome has emerged as a potent modulator of therapeutic outcome. Accumulating evidence suggests that the microbiota can markedly influence the efficacy of both chemotherapeutic and immunotherapeutic agents (14, 15). Although correlative studies abound, the mechanistic circuitry linking specific microbes, their metabolites, and the pharmacology of anticancer agents remains incompletely mapped. Bridging this knowledge gap is essential if microbiome-based interventions are to be moved from bench to bedside.

This review focuses on synthesizing recent advances in molecular studies that delineate how the gut microbiome modulates anticancer drug resistance. We will examine key mechanistic insights, including microbial regulation of drug metabolism and bioavailability, immunomodulatory effects, alteration of the tumor microenvironment, and metabolite-mediated signaling pathways, offering an integrated perspective on the microbiome as a targetable determinant of cancer therapy resistance. Among these interactions, microbiome-driven drug resistance has emerged as a central yet insufficiently characterized determinant of therapeutic failure, warranting systematic mechanistic dissection.

## 2 The gut microbiome and cancer therapy

### 2.1 Bidirectional interactions between chemotherapy and the gut microbiome

#### 2.1.1 Chemotherapeutic agents disrupt gut microbiota homeostasis

Chemotherapy not only targets rapidly proliferating tumor cells but also profoundly perturbs gut microbiota composition (16). Accumulating evidence from both animal models and clinical studies indicates that commonly used chemotherapeutic agents, such as 5-fluorouracil (5-FU) (17), methotrexate (18, 19), and gemcitabine (20), reduce microbial diversity and alter community structure. These changes are frequently characterized by depletion of beneficial commensals, particularly Firmicutes and Actinobacteria, alongside enrichment of potentially pathogenic or antibiotic-resistant taxa, including Proteobacteria (21).

Chemotherapy-induced dysbiosis is increasingly recognized as a contributor to gastrointestinal toxicity and systemic immune dysregulation. By disrupting microbial homeostasis, chemotherapeutic agents may indirectly reshape the intestinal immune landscape, thereby influencing treatment tolerance and downstream therapeutic outcomes.

#### 2.1.2 The gut microbiome modulates chemotherapy efficacy and toxicity

Beyond being a passive target of chemotherapy, the gut microbiome actively influences the efficacy and toxicity of chemotherapeutic agents (22). These effects are mediated primarily through microbial drug metabolism and microbiome-driven immune modulation, resulting in heterogeneous host responses to treatment.

##### 2.1.2.1 Microbial enzymatic inactivation of chemotherapy

One major mechanism by which gut microbes affect chemotherapy efficacy is direct enzymatic inactivation of drugs. A representative example is 5-FU, the primary first-line chemotherapeutic agent for colorectal cancer, which can be inactivated by *Escherichia coli* through conversion to dihydrofluorouracil (DHFU) via a bacterial preTA operon encoding a functional homolog of human dihydropyrimidine dehydrogenase (DPD). This microbial biotransformation reduces intracellular accumulation of active 5-FU metabolites, thereby attenuating DNA/RNA damage and contributing to chemoresistance (23). Similarly, gemcitabine resistance has been associated with intratumoral Gammaproteobacteria expressing long-form cytidine deaminase (CDD), which enzymatically converts gemcitabine into its inactive metabolite 2′,2′-difluorodeoxyuridine. This microbial inactivation reduces DNA replication stress and weakens gemcitabine-induced tumor cell apoptosis (24). In addition, bacterial β-glucuronidase (β-GUS) reactivates the inactive SN-38-glucuronide metabolite to cytotoxic SN-38, thereby intensifying the adverse effects of irinotecan (25). These findings highlight a conserved strategy whereby gut microbes alter chemotherapy efficacy and safety through direct metabolic interference with drug availability.

##### 2.1.2.2 Microbiota-mediated enhancement or resistance to chemotherapy

In contrast to drug inactivation, certain commensal bacteria enhance chemotherapy efficacy by modulating host antitumor immunity. Cyclophosphamide provides a well-characterized example, as its therapeutic activity partially depends on microbiome-driven immune priming. The translocation of specific Gram-positive bacteria, including *Enterococcus hirae* and *Lactobacillus johnsonii*, into secondary lymphoid organs stimulates dendritic cell maturation and promotes Th1/Th17 polarization, thereby enhancing CD8^+^ effector T-cell responses and increasing the intratumoral effector T cell-to-regulatory T cell ratio (26–28). Collectively, these observations highlight the dual role of the gut microbiome in chemotherapy, acting either as a barrier to treatment by inactivating drugs or as a facilitator of efficacy through immune activation (**Table 1**).

**Table 1:** Summary of the mechanisms of the gut microbiome and chemotherapeutic agents.

Drug	Cancer type	Microbiome	Core mechanism	Evidence level
5-FU	Gastrointestinal tumors	*Escherichia coli*	Enzymatic inactivation: DPD-like activity (29).	Preclinical
Gemcitabine	Pancreatic ductal adenocarcinoma	Gammaproteobacteria	Drug inactivation: CDD activity (24).	Preclinical
Irinotecan	Colorectal cancer	β-glucuronidase-producing bacteria	Toxic reactivation: β-GUS-driven SN38 regeneration (25).	Clinical
Oxaliplatin	Colorectal cancer	*Fusobacterium nucleatum*	Activation of TLR4-MYD88-NF-κB pathway (30, 31)	Preclinical and Clinical
Cyclophosphamide	Various (breast, lymphoma)	*Lactobacillus johnsonii/Enterococcus hirae*	Immunomodulation: Elevate the ratio of CD4+ T cells to regulatory T cells (28).	Preclinical
Methotrexate	various hematologic and solid tumors	*Variovorax paradoxus*	Toxicity mitigation: producing carboxypeptidase G2 (CPG2) (32).	Preclinical

### 2.2 Interactions between the gut microbiome and immunotherapy

Increasing evidence indicates that the gut microbiome is a critical determinant of host responsiveness to cancer immunotherapy, particularly immune checkpoint inhibitors (ICIs). Both microbial composition and microbiota-derived metabolites shape antitumor immunity and influence therapeutic efficacy as well as immune-related adverse events (33–35).

#### 2.2.1 The gut microbiome directly affects the efficacy of immunotherapy

Specific gut microbes have been consistently associated with improved responses to programmed death 1/programmed death-ligand 1 (PD-1/PD-L1) and Cytotoxic T-lymphocyte-associated antigen 4 (CTLA-4) blockade across cancer types (36–41). Among these, *Akkermansia muciniphila* (Akk) and *Bifidobacterium* species have been associated with improved responses to ICIs in several preclinical and clinical studies. Their presence correlates with enhanced T cell infiltration, improved antigen presentation, and prolonged progression-free survival (PFS) and overall survival (OS) in certain patient cohorts (42, 43). However, these effects appear to be highly context-dependent and may vary according to host immune status, dietary background, antibiotic exposure, tumor type, and baseline microbiota composition.

Mechanistically, these microbes promote a more immunostimulatory tumor microenvironment by reinforcing dendritic cell function and supporting effector T cell responses. Together, these findings suggest that gut microbiota composition may represent a potentially modifiable factor associated with immunotherapy responsiveness, although the reproducibility and causal relationships remain incompletely understood across different clinical settings.

#### 2.2.2 Microbial metabolites modulate immune responses

In addition to microbial composition, gut microbiota-derived metabolites play a central role in shaping host immunity during immunotherapy. Key metabolite classes include short-chain fatty acids (SCFAs), tryptophan derivatives, and secondary bile acids, which regulate immune cell function through metabolic and receptor-mediated pathways (3).

SCFAs can engage with G protein-coupled receptors 41 and 43 or inhibit histone deacetylases to produce immunotherapeutic effects (44). SCFAs such as butyrate can enhance antitumor immunity through multiple mechanisms, including HDAC inhibition and activation of G protein-coupled receptors such as GPR41, GPR43, and GPR109A. These pathways promote CD8^+^ T-cell metabolic fitness, cytokine production, and intratumoral infiltration, thereby improving responsiveness to immune checkpoint blockade (45, 46). Tryptophan metabolites exert context-dependent effects, with kynurenine promoting immunosuppression (47), while indole derivatives enhance antitumor immunity (48).In contrast, secondary bile acids such as deoxycholic acid (DCA) suppress CD8^+^ T cell effector function, thereby dampening immunotherapy efficacy (49).

However, the immunomodulatory effects of microbiota-derived metabolites remain highly context-dependent and are influenced by multiple host and environmental factors. Although Akk, *Bifidobacterium* species, and SCFAs have been associated with improved responses to ICIs in several studies, conflicting findings have also been reported across different cohorts and experimental settings. Variability in dietary background, antibiotic exposure, tumor type, host genetics, baseline microbiota composition, and treatment regimen may all contribute to inconsistent therapeutic outcomes. In addition, substantial differences between murine and human microbiomes limit the translational reproducibility of preclinical findings. Technical limitations, including sampling heterogeneity, sequencing platform variability, and the limited taxonomic and functional resolution of 16S rRNA sequencing, further complicate cross-study comparisons and causal interpretation. Therefore, future studies integrating longitudinal multi-omic profiling and mechanistic validation are required to better define causal microbiome-associated determinants of immunotherapy response and resistance.

Taken together, microbial metabolites represent a critical interface between the gut microbiome and host immune regulation, contributing to both responsiveness and resistance to immunotherapy.

### 2.3 Influence of gut microbiome on other anticancer drug treatments

While most research has focused on chemotherapy and immunotherapy, the gut microbiome may also influence responses to targeted and endocrine therapies. Recent studies have shown that the gut microbiome, as well as gut microbiome metabolites such as bile acids, SCFAs, and Trimethylamine N-oxide (TMAO), affect EGFR, VEGF, and KRAS targets that are frequently mutated in a variety of cancers (50). Additionally, there is evidence that the gut microbiome may indirectly influence the efficacy of endocrine therapy by regulating the metabolic state of the host. For example, the composition of the gut microbiome may affect estrogen metabolism, thus potentially affecting estrogen-dependent tumors such as breast cancer (51).

### 2.4 Linking the intratumoral microbiome to gut microbiota in anticancer resistance

The gut microbiota influences anticancer therapy mainly through certain mechanisms such as systemic metabolite production, immune modulation, and microbial translocation (52). In contrast, the intratumoral microbiome resides directly within tumor tissues, where it metabolizes drugs *in situ*, activates survival pathways, and locally reprograms antitumor immunity (53). Anticancer drugs, especially chemotherapeutics, disrupt gut homeostasis, leading to dysbiosis, barrier damage, and increased permeability (54). Viable bacteria and microbial products including lipopolysaccharide and bacterial DNA translocate across the intestinal epithelium, enter the circulation, and eventually seed distant tumor sites, thereby establishing or replenishing the intratumoral microbiome (55). Once established, intratumoral microbes directly reshape the local immune landscape by activating TLR and NF-κB signaling, driving pro-inflammatory cytokine production, recruiting immunosuppressive cells, and impairing effector T cell function. Simultaneously, these microbes directly modulate drug efficacy through enzymatic inactivation or by upregulating anti-apoptotic proteins (53). In summary, chemotherapy-induced gut dysbiosis establishes a tissue-specific intratumoral microbiome via the gut-to-tumor translocation axis. This tumor-resident microbiome then acts as an independent local driver of anticancer resistance through immune suppression and direct drug metabolism.

**Figure 1:**
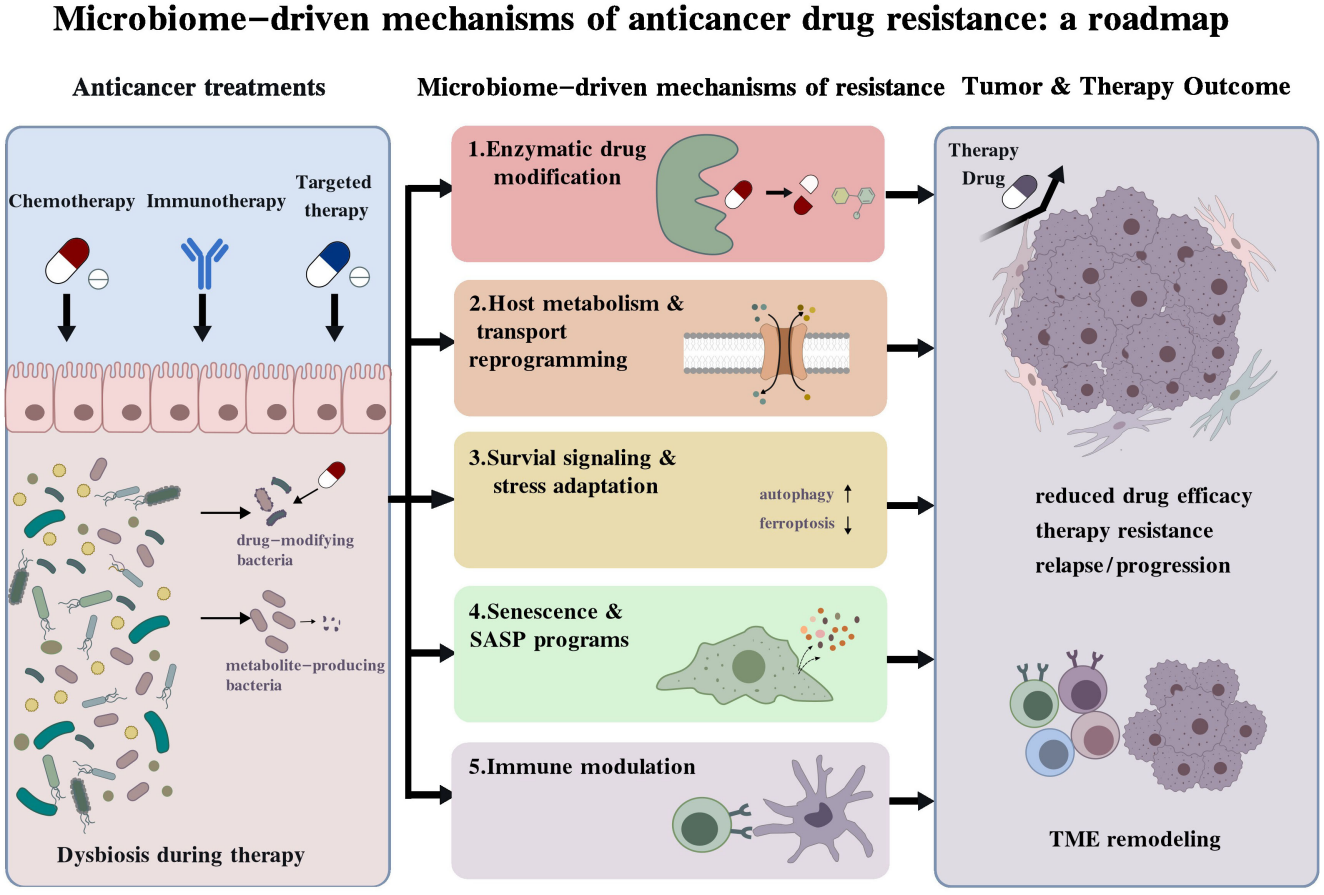
Microbiome-driven mechanisms of anticancer drug resistance: a roadmap. Anticancer therapies, including chemotherapy, immunotherapy, and targeted therapy, disrupt gut microbial homeostasis and induce dysbiosis. In turn, dysbiotic microbiota promote therapeutic resistance through five interconnected mechanisms: enzymatic drug modification causing inactivation or toxicity reactivation; reprogramming of host drug metabolism and transport, including cytochrome P450 remodeling and ABC/SLC transporter imbalance; activation of pro−survival pathways such as PI3K−AKT and NF−κB, along with enhanced autophagy and suppressed ferroptosis; induction of therapy−associated senescence and the senescence−associated secretory phenotype SASP that facilitates tumor escape; and modulation of antitumor immunity leading to impaired antigen presentation, T cell dysfunction, and immune evasion. Together, these mechanisms remodel the tumor microenvironment, reduce drug efficacy, and drive disease progression.

These findings highlight the broader relevance of the microbiome in anticancer therapy and lay the foundation for exploring microbiome-driven resistance mechanisms (**Figure 1**).

## 3 Mechanisms of microbiome-induced anti-cancer drug resistance

### 3.1 Microbial enzymatic modification of chemotherapeutic drugs

One of the most direct mechanisms by which the gut microbiome induces anticancer drug resistance is through microbial enzymatic modification of chemotherapeutic agents. Certain intestinal bacteria possess enzymes capable of metabolizing drugs into inactive or less effective forms, thereby reducing their bioavailability and therapeutic efficacy.

A representative example is 5-FU, which can be inactivated by *Escherichia coli* via enzymes encoded by the preTA operon, functionally analogous to human DPD. This microbial conversion of 5-FU into DHFU diminishes drug activity and contributes to chemoresistance (30). Similar preTA-like mechanisms have also been identified in other gut commensals, such as *Anaerostipes hadrus* (56), *Eubacterium siraeum*, and *Citrobacter* spp., indicating that 5-FU inactivation may be a conserved microbial strategy (57).

Another well-characterized case involves gemcitabine, which is metabolized into an inactive form by bacterial cytidine deaminase, predominantly expressed by Gammaproteobacteria. This enzymatic degradation has been linked to therapeutic failure in pancreatic and colorectal cancers (24, 58). In contrast to drug inactivation, microbial metabolism may also exacerbate toxicity, as exemplified by irinotecan. Bacterial β-glucuronidase reactivates the inactive metabolite SN-38-glucuronide in the gut, increasing local concentrations of active SN-38 and thereby intensifying gastrointestinal side effects that limit treatment tolerability (25).

Collectively, these findings illustrate that microbial enzymatic activity represents a major barrier to effective chemotherapy by directly altering drug stability, activity, and toxicity.

### 3.2 Microbiota-mediated regulation of host drug metabolism and transport

Beyond direct drug modification, the gut microbiome indirectly influences anticancer drug resistance by regulating host drug-metabolizing enzymes and transporters (59). Microbial metabolites modulate hepatic and intestinal biotransformation pathways, thereby reshaping systemic drug exposure and contributing to therapeutic resistance.

Gut microbiota-derived metabolites, including bile acids, short-chain fatty acids, and tryptophan derivatives, activate nuclear receptors such as pregnane x receptor (PXR) and aryl hydrocarbon receptor (AHR), thereby reprogramming host drug metabolism. Activation of these pathways reshapes cytochrome P450-mediated drug metabolism, altering the bioavailability and efficacy of multiple chemotherapeutic agents. Evidence from germ-free animal models further underscores the dependence of host drug-metabolizing capacity on microbial cues (60).

In parallel, microbial metabolites regulate drug transporter expression, particularly members of the ATP-binding cassette (ABC) and solute carrier (SLC) families. Secondary bile acids activate nuclear receptor signaling, leading to upregulation of efflux transporters such as ABCB1 and ABCC1 and enhanced drug extrusion. Additionally, butyrate has been shown to induce P-glycoprotein (P-gp) expression through the inhibition of histone deacetylase (HDAC) (61). Microbiome-driven metabolic reprogramming may further suppress SLC transporter expression, impairing drug uptake and limiting therapeutic efficacy. This mechanism is particularly relevant for hydrophilic chemotherapeutic and targeted agents that rely on transporter-mediated cellular entry.

Together, microbiome-driven modulation of host metabolism and transport constitutes an indirect but highly influential mechanism of anticancer drug resistance.

### 3.3 Activation of drug resistance-associated signaling pathways

Emerging evidence indicates that specific gut microbes and their metabolites promote drug resistance by activating tumor-intrinsic survival and stress-response signaling pathways. These pathways converge to enhance cell survival, inhibit cell death, and support therapy escape.

Several bacteria, including *Fusobacterium nucleatum* and *Clostridium perfringens*, have been shown to activate PI3K-AKT, NF-κB, and TLR4/MyD88 signaling cascades. In colorectal cancer, *Fusobacterium nucleatum* has been associated with oxaliplatin resistance through activation of the TLR4/MYD88 signaling axis, which subsequently enhances autophagy and upregulates glutathione peroxidase 4 (GPX4) expression, thereby suppressing ferroptotic cell death and promoting tumor cell survival during chemotherapy (62). Separately, *Clostridium perfringens* activates the TLR4/MyD88 pathway, leading to selective loss of autophagy-related microRNAs and subsequent induction of autophagy, which compromises the efficacy of oxaliplatin and 5-FU (30, 31). Furthermore, lipopolysaccharide (LPS) from Gram-negative bacteria binds to Toll-like receptor 4 (TLR4), activating both the PI3K-AKT-mTOR and MyD88-MAPK signaling cascades, which converge to promote NF-κB-mediated transcription (63). These observations underscore that microbiota-induced signaling rewiring establishes a tumor microenvironment conducive to therapeutic resistance.

### 3.4 Microbiome-driven senescence and SASP-associated resistance

Chemotherapy and radiotherapy can induce cellular senescence in tumor cells, which in turn promotes drug resistance through the senescence-associated secretory phenotype (SASP). SASP is characterized by the secretion of pro-inflammatory cytokines, growth factors, and matrix-remodeling enzymes that support tumor progression and therapy escape (64). Recent studies reveal that certain bacteria, notably *Fusobacterium nucleatum* and *Clostridium perfringens*, exacerbate SASP-mediated resistance by invading senescent tumor cells and activating DNA damage response pathways. In esophageal squamous cell carcinoma, bacterial-induced SASP enhances tumor survival and chemoresistance, highlighting a previously underappreciated interaction between microbial infection, senescence, and treatment failure (65). These findings suggest that microbiome-driven modulation of senescence programs represents an additional layer of resistance regulation (66).

### 3.5 Microbiome-mediated immune modulation and resistance to immunotherapy

The gut microbiome also contributes to resistance against cancer immunotherapy by reshaping host immune responses. Dysbiosis can impair antigen presentation, alter T cell differentiation, and suppress antitumor immunity, thereby limiting the efficacy of ICIs.

Current research on immunotherapy resistance due to gut microbiota has focused on Akk (67, 68) and *Fusobacterium nucleatum* (69), among others. Specific microbes appear to exert context-dependent effects. Akk has been associated with improved responses to PD-1 blockade in both murine tumor models and clinical NSCLC cohorts, potentially through IL-12-dependent dendritic cell activation and enhanced CD8^+^ T-cell recruitment into the tumor microenvironment (43). Regarding *Fusobacterium nucleatum*, its derived succinic acid suppressed the cGAS-interferon-β pathway, consequently dampening the antitumor response by limiting CD8^+^ T cell trafficking to the tumor microenvironment (TME) *in vivo* (69). In addition, microbiological analyses showed that *Parabacteroides distasonis* and *Bacteroides vulgatus* were in higher abundance in anti-PD-1 blocker responders than in non-responders in the clinic. Thus, if gut microbiota dysbiosis leads to a decrease in the abundance of these two microbiota, it may be associated with resistance to anti-PD-1 blockers (70). Collectively, these studies highlight that microbiome-mediated immune modulation is a critical determinant of both responsiveness and resistance to cancer immunotherapy.

### 3.6 An integrative feedback network of microbiome-driven drug resistance

The discrete mechanisms above, including enzymatic modification, transport regulation, signaling activation, senescence, and immune modulation, do not function independently but rather as an interconnected feedback network. Therapy-induced dysbiosis compromises intestinal epithelial integrity, enabling microbial metabolites and pathogen-associated molecular patterns to translocate into the circulation and the tumor microenvironment. Such microbial products incite local and systemic inflammation, which reshapes antitumor immunity by expanding immunosuppressive populations and dampening effector T cell function. Simultaneously, microbial metabolites directly reprogram tumor cell metabolism and activate pro-survival pathways like PI3K-AKT and NF-κB, thereby promoting therapy evasion. Persistent inflammation and metabolic alterations further erode barrier integrity, closing a positive feedback loop that sustains dysbiosis and resistance. Additionally, chemotherapy-induced senescent tumor cells acquire a SASP that releases pro-inflammatory cytokines, further destabilizing microbial homeostasis and reinforcing the cycle.

## 4 Strategies to overcome microbiome-related anti-cancer drug resistance

### 4.1 Modulation of the gut microbiome to enhance therapeutic efficacy

Given the central role of the gut microbiome in shaping anticancer drug responses, multiple strategies have been explored to modulate microbial composition and function to overcome therapy resistance. These approaches primarily aim to restore microbial balance, enhance antitumor immunity, or reduce microbial-mediated drug inactivation.

Dietary intervention, probiotics, prebiotics, synbiotics, and postbiotics represent noninvasive strategies to reshape the gut microbiota. Probiotic supplementation with beneficial taxa such as *Bifidobacterium* (42), *Lactobacillus* (71, 72), and Akk (43) have been associated with improved responses to ICIs by enhancing T cell-mediated immunity.

Prebiotics, including inulin (73) and pectin (74), selectively promote the growth of beneficial microbes and strengthen intestinal barrier function, thereby indirectly supporting antitumor immune responses. Synbiotics represent a synergistic combination of probiotics and prebiotics, designed to optimize the benefits of both. In addition, postbiotics such as SCFAs directly modulate immune cell function and inflammation (45, 46, 75).

However, several studies have reported that probiotic supplementation fails to improve responses to chemotherapy or immunotherapy. In a cohort of melanoma patients, probiotic use was associated with reduced responsiveness to anti−PD−1 therapy, and corresponding animal models exhibited increased tumor burden following probiotic administration (76). Additional evidence indicates that probiotics can disrupt gut microbial homeostasis or impede post−antibiotic microbiota reconstitution, thereby potentially attenuating antitumor immunity. Moreover, meta−analyses have concluded that the evidence supporting an extension of progression−free or overall survival with probiotics remains inconclusive and demonstrates substantial cross−study heterogeneity (77).

Despite their theoretical promise, microbiome-targeted interventions exhibit substantial context-dependent variability and are influenced by baseline microbiota composition, dietary background, cancer type, antibiotic exposure, and treatment regimen. Several studies have reported inconsistent or even adverse effects following probiotic supplementation, suggesting that indiscriminate microbiome modulation may not universally benefit all patients. Therefore, personalized and mechanism-guided microbiome interventions are likely required to maximize therapeutic efficacy while minimizing unintended immune or metabolic consequences.

### 4.2 Fecal microbiota transplantation and antibiotic intervention

Fecal microbiota transplantation (FMT) represents a more direct strategy to reprogram the gut microbiome. Initially developed for recurrent *Clostridioides difficile* infection (78), FMT has shown potential to restore responsiveness to ICIs in patients with melanoma (79), colorectal cancer (80), and other solid tumors (81). However, the efficacy of FMT remains inconsistent across tumor types and patient cohorts, and most current evidence is derived from relatively small-scale and early-phase clinical studies. By increasing microbial diversity and introducing beneficial taxa, FMT may contribute to partially overcoming microbiome-associated immunotherapy resistance (82). However, FMT faces substantial challenges, including donor selection, safety concerns, long-term stability, and regulatory barriers.

Beyond these logistical issues, FMT carries documented risks, including transmission of multidrug-resistant pathogens such as extended-spectrum beta-lactamase-producing *Escherichia coli* leading to sepsis (83) and potential induction of proinflammatory dysbiosis that may promote tumor progression (84). Although recent trials of FMT combined with immunotherapy reported no grade 4 or grade 5 toxicity, grade 3 immune-related adverse events occurred in up to 50% of patients (85).

Moreover, the therapeutic efficacy of microbiome-based interventions remains variable across studies and clinical settings. While encouraging responses to FMT and probiotic supplementation have been reported in certain cancers, several studies have demonstrated inconsistent outcomes or even impaired immunotherapy efficacy following probiotic administration. Such discrepancies likely reflect inter-individual microbiota heterogeneity, donor-dependent effects, dietary differences, antibiotic exposure, and variability in microbial engraftment efficiency. Additionally, most current clinical evidence is derived from relatively small-scale and early-phase studies, limiting the generalizability of existing findings. Therefore, standardized protocols, large prospective cohorts, and mechanistic validation studies are still required before microbiome-based interventions can be broadly implemented in clinical oncology.

Antibiotics have also been explored to eliminate bacteria that inactivate drugs and enhance the efficacy of chemotherapy. For example, ciprofloxacin can inhibit the growth of certain drug-resistant bacteria (e.g., *Aspergillus*), thereby enhancing the effects of chemotherapeutic drugs (e.g., gemcitabine) (86). While selective antibiotic use may improve outcomes in specific settings, broad-spectrum antibiotic exposure often induces dysbiosis and is associated with impaired immunotherapy responses. In particular, antibiotic administration within 30–60 days prior to ICIs initiation consistently correlates with worse PFS and OS, likely due to depletion of beneficial taxa such as Akk and *Bifidobacterium* spp (87, 88). Therefore, antibiotic intervention remains a double-edged sword, requiring cautious and context-specific application.

### 4.3 Development of therapies less susceptible to microbial interference

An alternative strategy to overcome microbiome-related resistance is the development of anticancer therapies that are less vulnerable to microbial modification. Nanotechnology-based drug delivery systems offer a promising solution by encapsulating chemotherapeutic agents within nanoparticles, thereby shielding them from microbial enzymes and enabling targeted release within the tumor microenvironment (89). Such systems can enhance drug accumulation, reduce off-target toxicity, and potentially bypass microbial-mediated inactivation. A recent study developed a lipid polymer nano-assembly based on PEG-distearoylphosphatidyl-ethanolamine (DSPE-PEG), which has shown significant tumor growth inhibition and enhanced immune cell activation in various chemotherapy-resistant animal tumor models (90).

**Figure 2:**
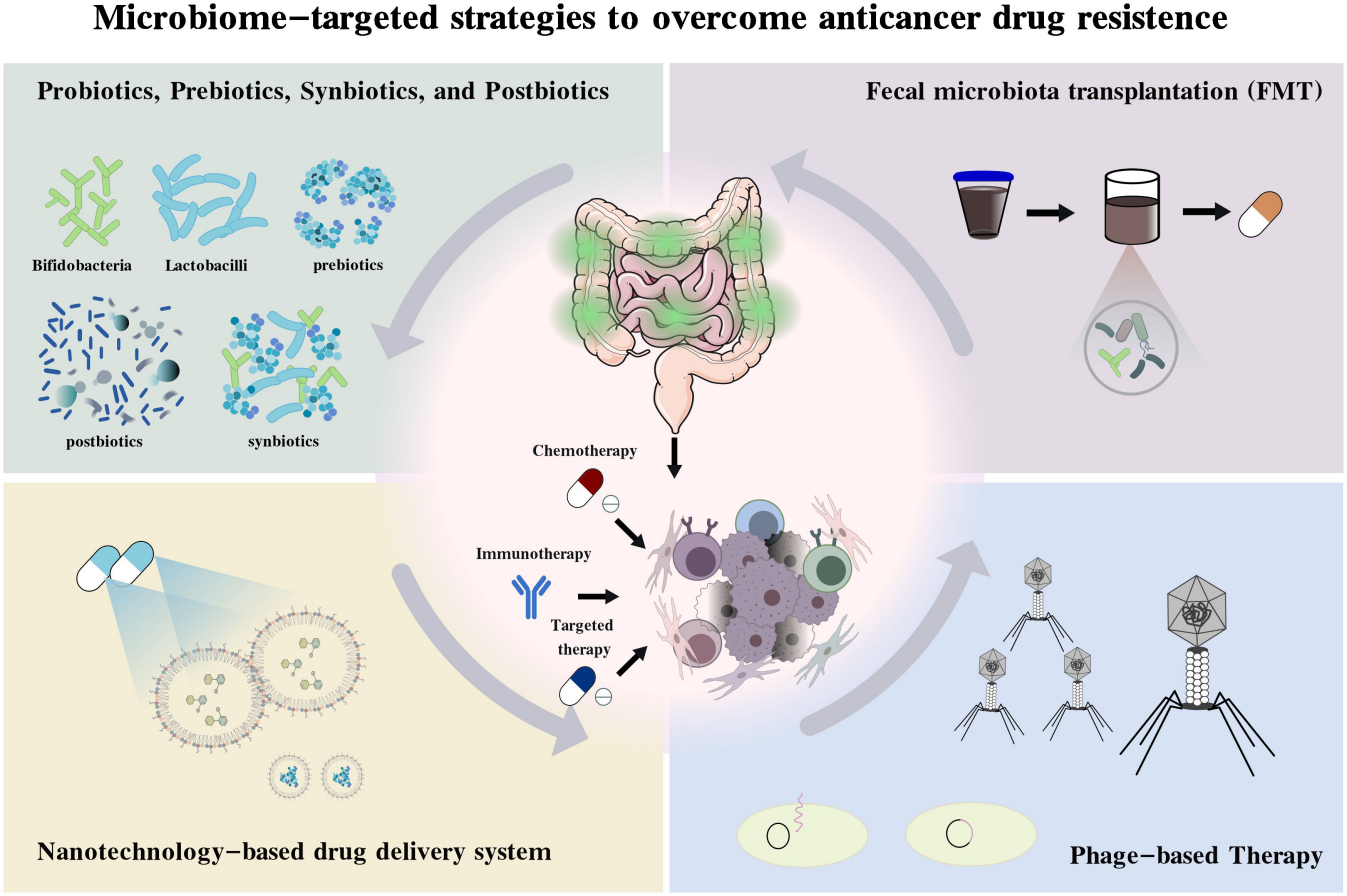
Microbiome-targeted strategies to overcome anticancer drug resistance. Intervention strategies include modulation of gut microbiota composition through dietary intervention, prebiotics, probiotics, synbiotics, and postbiotics; global microbiome reprogramming via fecal microbiota transplantation (FMT); selective elimination or functional inhibition of drug−modifying microbes and their enzymes; and advanced drug delivery systems that protect anticancer agents from microbial interference.

In parallel, phage-based therapies have emerged as an innovative approach to target tumor-associated or drug-modifying bacteria selectively. Phages can be engineered to eliminate specific bacterial populations, deliver therapeutic payloads, or modulate antitumor immunity (91). Although preclinical studies suggest considerable potential, clinical translation remains in its early stages, with challenges related to specificity, safety, and scalability. Collectively, these microbiome-targeted strategies highlight a mechanism-guided framework for overcoming therapeutic resistance, as summarized in **Figure 2**.

## 5 Challenges and future perspectives

Accumulating evidence positions the gut microbiome as a critical determinant of anticancer drug resistance, operating at the intersection of drug metabolism, host immunity, and tumor microenvironment remodeling. Rather than acting through isolated mechanisms, microbiome-driven resistance emerges as a multilayered process in which microbial metabolism, host signaling pathways, and immune responses converge to shape therapeutic outcomes.

Despite rapid advances, several key challenges remain. Firstly, most current studies are correlative, and establishing causal relationships between specific microbial taxa, metabolites, and drug resistance mechanisms remains difficult. Secondly, significant microbiota heterogeneity and individual variations complicate studies, as the microbiota is influenced by genetics, diet, antibiotics, and other factors, and its interactions may differ across tumor types, stages, and treatments, hindering the identification of universal key microbial biomarkers or intervention strategies. Finally, limitations in research models pose a barrier: commonly used *in vitro* or mouse models differ substantially from human microbiota, and standardized platforms for studying human microbiota-tumor interactions are lacking, significantly impeding clinical translation.

Addressing these challenges requires integrated efforts across multiple fronts. Technologically, the field must develop tools to elucidate spatiotemporal dynamics and causality; integrating single-cell multi-omics with artificial intelligence will be key to moving from correlation to mechanistic understanding. In model development, patient-derived organoid-autologous microbiota co-culture platforms offer a promising direction to better mimic human host-microbe interactions. For clinical translation, priority should be given to prospective studies that identify robust microbial biomarkers, including metagenomic signatures and metabolomic profiles, to enable patient stratification and prediction of therapeutic responses. Longitudinal microbiome profiling captures treatment-induced dynamic shifts, facilitating early detection of resistance-associated changes. Artificial intelligence-driven models integrating multi-omic data can further guide therapy selection and support microbiome-based companion diagnostics. Finally, personalized microbiome interventions tailored to baseline microbial composition, such as engineered bacterial therapies, represent a promising approach to overcome resistance. Fostering interdisciplinary collaboration and international standardized data platforms will be crucial to translating these concepts into tangible clinical strategies.

Although this review focuses on bacteria, emerging evidence suggests that non-bacterial microorganisms, including the mycobiome, virome, and bacteriophages, may also influence anticancer drug resistance. Fungi such as *Candida albicans* can modulate tumor-associated inflammation, while gut viruses and phages reshape bacterial community structure and function through inter-kingdom interactions. Phages may even directly affect host immune signaling. Incorporating multi-kingdom metagenomic analyses into future studies will be essential to fully understand microbiome-driven resistance and to identify novel therapeutic opportunities.

Ultimately, incorporating microbiome-aware frameworks into precision oncology has the potential to improve therapeutic durability and overcome drug resistance. By viewing the microbiome as an integral component of the host-tumor-drug axis, future studies may unlock new opportunities to personalize cancer treatment and enhance clinical outcomes.
